# Expansion and persistence of antibiotic-specific resistance genes following antibiotic treatment

**DOI:** 10.1080/19490976.2021.1900995

**Published:** 2021-03-28

**Authors:** Kang Kang, Lejla Imamovic, Maria-Anna Misiakou, Maria Bornakke Sørensen, Yoshitaro Heshiki, Yueqiong Ni, Tingting Zheng, Jun Li, Mostafa M. H. Ellabaan, Marta Colomer-Lluch, Anne A. Rode, Peter Bytzer, Gianni Panagiotou, Morten O.A. Sommer

**Affiliations:** a Novo Nordisk Foundation Center for Biosustainability,Technical University of Denmark, Lyngby, Denmark; b Leibniz Institute for Natural Product Research and Infection Biology, Systems Biology and Bioinformatics - Hans Knoell Institute, Jena, Germany; c Kadoorie Biological Sciences Building, School of Biological Sciences, the University of Hong Kong, Hong Kong, S. A. R. China; d Department of Infectious Diseases and Public Health, Colleague of Veterinary Medicine and Life Sciences, City University of Hong Kong, Hong Kong, S.A.R. China; e School of Data Science, City University of Hong Kong, Hong Kong, S. A. R. China; f Department of Medicine, Zealand University Hospital - Køge, Køge, Denmark; g Department of Clinical Medicine, University of Copenhagen, Copenhagen, Denmark; h Department of Pharmacology and Pharmacy, the University of Hong Kong, Hong Kong, S. A. R. China

**Keywords:** Gut microbiome, virome, phage, mobile element, antibiotic resistance, antibiotic treatment

## Abstract

Oral antibiotics are commonly prescribed to non-hospitalized adults. However, antibiotic-induced changes in the human gut microbiome are often investigated in cohorts with preexisting health conditions and/or concomitant medication, leaving the effects of antibiotics not completely understood. We used a combination of omic approaches to comprehensively assess the effects of antibiotics on the gut microbiota and particularly the gut resistome of a small cohort of healthy adults. We observed that 3 to 19 species per individual proliferated during antibiotic treatment and Gram-negative species expanded significantly in relative abundance. While the overall relative abundance of antibiotic resistance gene homologs did not significantly change, antibiotic-specific gene homologs with presumed resistance toward the administered antibiotics were common in proliferating species and significantly increased in relative abundance. Virome sequencing and plasmid analysis showed an expansion of antibiotic-specific resistance gene homologs even 3 months after antibiotic administration, while paired-end read analysis suggested their dissemination among different species. These results suggest that antibiotic treatment can lead to a persistent expansion of antibiotic resistance genes in the human gut microbiota and provide further data in support of good antibiotic stewardship.

**Abbreviation**: ARG – Antibiotic resistance gene homolog; AsRG – Antibiotic-specific resistance gene homolog; AZY – Azithromycin; CFX – Cefuroxime; CIP – Ciprofloxacin; DOX – Doxycycline; FDR – False discovery rate; GRiD – Growth rate index value; HGT – Horizontal gene transfer; NMDS – Non-metric multidimensional scaling; qPCR – Quantitative polymerase chain reaction; RPM – Reads per million mapped reads; TA – Transcriptional activity; TE – Transposable element; TPM – Transcripts per million mapped reads

## Introduction

The gut microbiota is a complex collection of bacteria, archaea, eukaryotic cells, and viruses. Humans and their gut microbiota maintain a dynamic equilibrium that is important for metabolic homeostasis, immune regulation, and pathogen susceptibility. Human host-microbiota symbiosis is affected by factors such as genetic background, diet, and drug treatment.^1^ In particular, antibiotic therapy can perturb the gut microbiota composition and potentially interfere with the optimal functioning of the gut microbiome in an individualized and time-dependent manner.^2,3^ Antibiotic treatment is associated with reduced microbiome diversity^4–6^ and development of infection with bacteria such as *Clostridium difficile*.^7,8^ Yet, approximately one-third of adults in the European Union received at least one course of oral antibiotics in 2015.^9^ Thus, antibiotic stress on the gut microbiome is common; however, its effects on the healthy human gut are still not completely understood.

Many studies that assessed the impact of antibiotics on gut bacterial communities were based on 16S rRNA gene amplicon sequencing.^6,7^ While such an assessment identifies the species composition, it cannot elucidate bacterial functions in the communities or determine changes in the repertoire of antibiotic resistance genes (ARGs).^6^ Even when the full functional potential of the community is evaluated with shotgun metagenomics, this assessment can be biased since DNA from bacteria killed by antibiotic treatment is still detected.^10^ Maurice *et al*. investigated the effect of xenobiotics on the active human gut microbiome *in vitro* by direct application of drugs to human stool samples. They reported that one-third of the gut microbiota was damaged cells.^2^ While this study moved beyond a survey of diversity to understand metabolic activities, the *in vitro* design did not account for the pharmacokinetic and pharmacodynamic properties of an antibiotic in the human body. A recent study reported that the gut microbiota is resilient to a short-term intervention of broad-spectrum antibiotics, with no clear temporal pattern in the overall relative abundance of ARGs.^3^ Because the study used an antibiotic cocktail, the antibiotic-specific responses of the gut microbiota and the long-lasting imprint on the resistome (the collection of ARGs in a microbiome) could not be determined and traced with high resolution. Combined metagenomics and metatrancriptomics analysis have been employed to investigate the effects of antibiotics on transcriptional activities of gut microbial communities of hospitalized individuals.^11^ However, in these studies, antibiotic effects on the gut might be confounded by factors such as hospital stay, preexisting health conditions, and/or concomitant medication.

The healthy human gut microbiome harbors diverse antibiotic resistance mechanisms.^12^ These include enzymes involved in drug inactivation or modification, efflux systems, or polymorphisms in antibiotic gene targets. Of concern is that antibiotic stress affects not only targeted species but the overall composition of microbial communities, leading to the accumulation of antibiotic resistance traits. For example, the macrolide resistance gene *ermB*, amplified by antibiotic treatment, was stable in gut bacterial population years after treatment.^13^ This result indicated that a resistance fraction in the population can be selected and amplified during and after treatment.^13^ Antibiotic stress can result in sensitive bacteria evolving resistance mechanisms by selecting for gene variants that confer higher resistance.^14,15^

Importantly, ARGs can also be transferred within and between species,^16^ particularly in complex microbial communities such as the gut.^17,18^ Horizontal gene transfer mediated by plasmids played a prominent role in the spread of antibiotic resistance within the same species or between different species.^19^ Furthermore, the human gut is colonized by diverse bacteria, the genomes of which can contain up to 20% of prophage DNA.^20,21^ Such phages can incorporate part of their host’s genetic material, including antibiotic resistance genes during generalized transduction, and could thus contribute to the rapid dissemination of resistance among bacteria. A metagenomic study on antibiotic alteration in mice showed enrichment in antibiotic resistance genes in the virome fraction 2 months after treatment.^22^ However, a subsequent study indicated that the phage-harbored reservoir of antibiotic resistance genes might have been overestimated due to loosening threshold levels used *in silico* detection of antibiotic resistance genes.^23^ Thus, the importance of phages as reservoirs of antibiotic resistance genes remains debated.

In this study, we investigated the effect of antibiotics on the composition and resilience of the healthy human gut microbiome. We combined metagenome, metatranscriptome, and virome sequencing for a comprehensive assessment of the living, active microbiota, and its associated phages and plasmids.

## Results

### Microbial community structure shifted during antibiotic treatment and recovered after treatment

To evaluate the effect of antibiotic treatment on the human gut microbiome, 10 healthy human volunteers were recruited and randomized to receive one in four different antibiotic courses or to be a control (**Study design, Methods**). Before, during, and after exposure to antibiotics, they provided a total of six stool samples (**Figure S1**). Four antibiotics were selected based on their clinical relevance and broad-spectrum activity against Gram-positive and/or Gram-negative organisms (**Table S1**). We evaluated the impact of antibiotic therapy on the microbiome using metagenomic, metatranscriptomic and viromic profiling of stool samples (**Data S1**).

**Figure 1.**
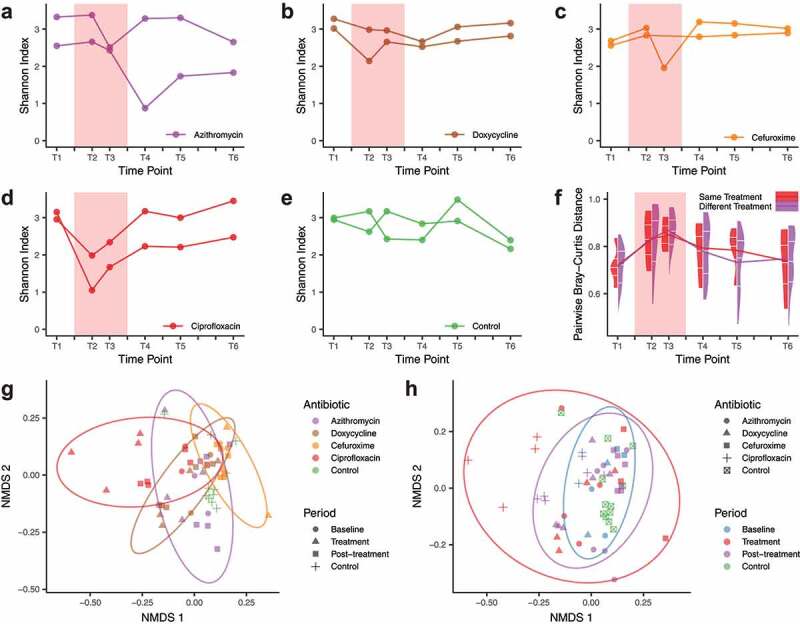
Microbial community diversity and dissimilarity

In the majority of samples, Bacteroides and Firmicutes were dominant at the phylum level (**Figure S2a**). In general, the richness and alpha-diversity measured as Shannon index dropped significantly when compared with the baseline levels (Wilcoxon signed-rank test, *p* = .03 and 0.007) (Figure 1a**-e**), and the inter-individual community dissimilarity increased (measured as pairwise Bray–Curtis distance, Wilcoxon signed-rank test, *p* = 1 × 10^−10^), regardless of administering the same antibiotic or two different ones (Figure 1f). Community diversity was recovered to baseline levels after ciprofloxacin (CIP)and cefuroxime (CFX) treatments (post-treatment *vs*. baseline, two-tailed paired t-test, *FDR* = 0.19 and 1.00) in line with previous studies.^3,6,24^ However, doxycycline (DOX) and azithromycin (AZY) post-treatment communities continued to have relatively low diversity (post-treatment *vs*. baseline, Wilcoxon signed-rank test, *p* = .03 and 0.03), especially for azithromycin (Shannon index reduction after 3 months: 20.2% for AZY-a and 28.2% for AZY-b). In a comparison of antibiotics, baseline species-level composition did not show significant differences (Adonis test, *p* = .41), but community composition during and after treatment differed significantly (Adonis test, *p* = .01) (Figure 1g**-h**). In spite of the small sample size, this result indicated that the alterations in community structures were specific to a given antibiotic and the post-treatment recovery of the community structure may also be influenced by the antibiotic type.

**a-e**. Alpha-diversity (Shannon index) perturbation during and after different antibiotic treatments (**a-d**) and in the control group (**e**). **f**. Pairwise Bray–Curtis distances between two individuals given the same treatment or two different treatments. The split violin plots demonstrate the distribution of the distances, with the 25%, 50%, and 75% quantiles marked with white ticks. The line graphs represent the mean values. In panels **a-f**, the red background indicates the antibiotic administration period. **g-h**. Community structures for the microbiomes of participants receiving different antibiotic treatments and control samples (T1 to T6). Non-metric multidimensional scaling (NMDS) was applied to Bray–Curtis distances (2D NMDS stress: 0.18). The samples were colored with different information: antibiotic groups (**g**) or treatment periods (**h**). The ellipses depict a confidence level of 90% for the samples with the same color. Antibiotic treatment shifted the community structure detectably during treatment, followed by a fast recovery in the post-treatment period.

**Figure 2.**
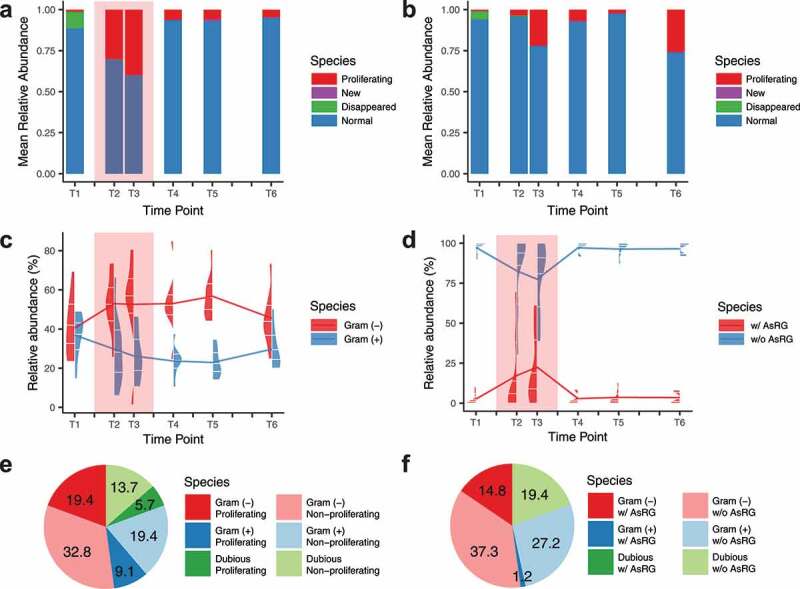
The relative abundances of different species categories in different antibiotic treatment periods

The long-term consequences of antibiotic treatment were captured at the species level 3 months after treatment. For the antibiotic-treated groups, 0 to 21 species (median: 3.5) with a relative abundance of 0 to 47.7% (median: 3.58%) at baseline were undetectable after treatment and did not recover 3 months after antibiotic treatment (Figure 2a **and Table S2)**. Except for AZY-b (baseline richness: 65, lower than all other baseline samples with a richness of 86 to 116), regardless of treatment or control, few new species (0 to 1) emerged during or after treatment, with maximum relative abundances of 0.88% during treatment (CFX-b) and 7.7% in post-treatment (CIP-b) (Figure 2a).

**a-b**. Mean relative abundance of species categories for eight individuals treated with antibiotics (**a**) and two control individuals (**b**). **c**. The relative abundance of Gram-positive and Gram-negative species. **d**. The relative abundance of species harboring antibiotic-specific resistance gene homologs (AsRGs). In panels **a-d**, the red background indicates the antibiotic administration period. In panels **c-d**, the split violin plots demonstrate the distribution of relative abundances, with the 25%, 50%, and 75% quantiles marked with white ticks. The line graphs represent the mean values. **e-f**. The relative abundance of Gram-positive and Gram-negative species in proliferating species (**e**) and species harboring AsRGs (**f**) during treatment.

To study species with strong competitive advantages during treatment, we focused on the species that proliferated during T2 and T3 (“proliferating species”) (Figure 2a**-b, S2b**, and **Table S2**), defined as 1) a less abundant species in baseline (relative abundance < 0.1%) reached 5% in relative abundance in T2 or T3, or 2) an abundant species in baseline (relative abundance ≥ 0.1%) increased by at least 10-fold relative abundance in T2 or T3. For each individual (including controls), 1 to 6 species (median: 4) were identified as proliferating species (**Table S2**). Among proliferating species, *Bacteroides caccae* was the most common one, shared by three individuals (**Figure S2b** and **Table S2**). Noticeably, the proliferating species had significantly predicted higher growth rates^25^ regardless of the treatment period (growth rate index value (GRiD) median: 1.2), compared with non-proliferating species (GRiD median: 1.0, Wilcoxon rank-sum test, *p* = 2 × 10^−10^) (**Figure S3**).

### Gram-negative species and carriers ofantibiotic-specific resistance gene homologs (AsRGs) proliferated during treatment

To further assess the competitive advantages of gut bacteria during antibiotic treatment, we classified all species as Gram-positive or Gram-negative.^26^ The relative abundance of Gram-negative species significantly increased from 40.5% at baseline to 53.0% during treatment (Wilcoxon signed-rank test, *p =* .02) (Figure 2c). Gram-negative species also had a higher probability of becoming a proliferating species. Of the 254 Gram-negative species, 12 species proliferated, with a relative abundance of 19.4% during treatment. In comparison, of the 554 Gram-positive species, only 12 species proliferated with an abundance of 9.1% (chi-square test, *p* = .043) (Figure 2e).

To study species-specific resistance profiles, we compiled a list of antibiotic-specific resistance gene homologs (AsRG) that can confer or contribute to clinically relevant resistance to antibiotics given to the study participants (**Table S3**) and assigned AsRGs to specific host species (see **Methods**). For each individual, 0 to 8 species (median: 2.5) were identified as AsRG carriers via a contig-species assignment procedure (species names aligned with the taxonomic profiles to get regarding relative abundances, see **Methods** and **Data S2** for details). Similar to the proliferating species, more Gram-negative species were identified as AsRG carriers (15/254 Gram-negative species with a relative abundance of 14.8% during treatment *vs*. 11/554 Gram-positive species with an abundance of 1.2%, chi-square test, *p* = .007) (Figure 2f). Noticeably, AsRG carriers had a higher tendency to proliferate than the species harboring no identified AsRGs (6/30 *vs*. 14/1140, unclassified species and proliferating phage excluded, Fisher test, *p* = 5 × 10^−6^). In general, AsRGs tended to have high copy numbers (**Figure S4a**) and multiple host species (**Figure S4b**) in the metagenomic assemblies. The relative abundance of AsRG carriers also increased from 3.0% to 21.1% during antibiotic treatment (Wilcoxon signed-rank test, *p* = .03) (Figure 2d) indicating that AsRGs confer a fitness advantage during antibiotic treatment.

### AsRGs have high transcriptional activity during antibiotic treatment

**Figure 3.**
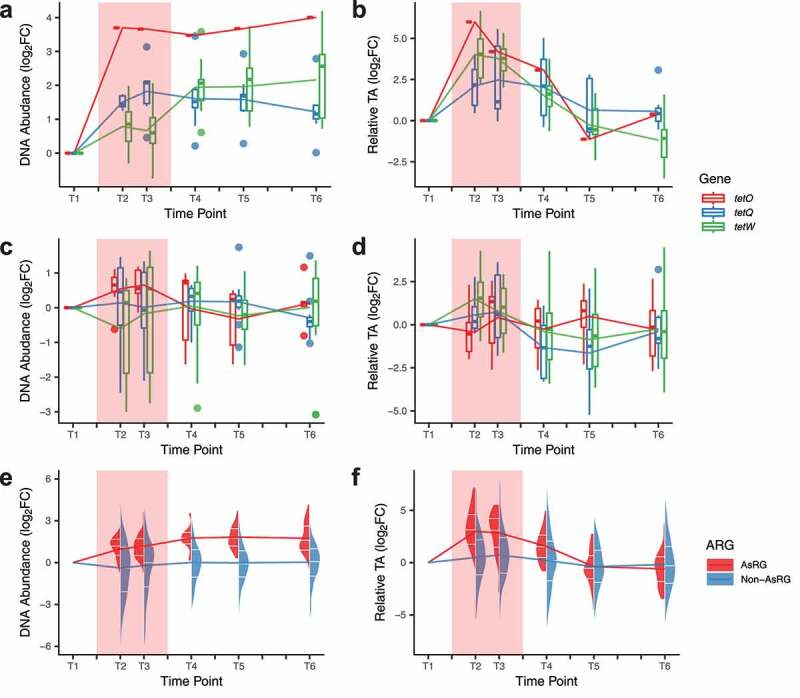
DNA relative abundance and transcriptional activity (TA) of AsRG

To determine if antibiotic treatment exerts selective pressure on the resistome as a whole, we analyzed the changes in ARGs for each antibiotic used in the study. Detected resistance genes did not show an overall increase in DNA relative abundance or transcriptional activity. However, specific trends in AsRGs were observed (**Figure S5)**. As the most prevalent gene family that could also be detected in the individuals not treated with tetracycline, ARGs conferring resistance to tetracycline antibiotics showed increased DNA relative abundance and high expression during doxycycline (DOX) treatment (Figure 3a**-b**). The induction of tetracycline resistance genes by doxycycline has been observed before and is in fact utilized for fine-tuning the expression of specific genes in eukaryotic cells.^27^ In contrast, no clear patterns in DNA abundance or transcriptional activity were observed during treatment with other antibiotics (Figure 3c**-d**). The DNA relative abundance of AsRGs significantly increased during treatment compared to non-AsRGs (median fold-change: 2.4 *vs*. 0.9, Wilcoxon rank-sum test, *p =* 4 × 10^−9^) (Figure 3e). AsRGs’s transcriptional activities were also exceptionally boosted during treatment (median fold-change from baseline: 9.5, Wilcoxon signed-rank test, *p =* 2 × 10^−8^) (Figure 3f) and largely outperformed non-AsRGs (median fold-change from baseline: 1.5, Wilcoxon rank-sum test, *p =* 2 × 10^−12^). Noticeably, while the transcriptional activity of AsRGs was reduced after treatment (Figure 3b and 3f) (no significant difference between post-treatment and baseline), the DNA relative abundance of AsRGs did not decline even 3 months after treatment (median fold-change from T1 to T6: 2.7, Wilcoxon signed-rank test, *p =* 2 × 10^−9^) (Figure 3a and 3e).

In all panels, the red background indicates the antibiotic administration period. **a-b**. DNA relative abundance (**a**) and transcriptional activity (**b**) of *tet* family genes as AsRGs for doxycycline treatment. DNA abundances were calculated by transcripts per million mapped reads (TPM). AsRGs’ transcriptional activities were calculated by RNA (TPM)/DNA (TPM). **c-d**. DNA relative abundance (**c**) and transcriptional activity (TA) (**d**) of *tet* family genes as non-AsRGs. **e-f**. DNA relative abundance (**e**) and transcriptional activity (**f**) of all AsRGs and non-AsRGs The DNA abundance or relative TA were normalized to log2 fold-change of baseline level in all panels. The split violin plots demonstrate the distribution of DNA abundances or TAs, with the 25%, 50%, and 75% quantiles marked with white ticks. The line graphs represent the mean values.

### Virome-encoded ARGs expanded after antibiotic treatment

To further understand the basis for AsRG expansion during antibiotic treatment, we performed virome sequencing and assembly (**Figure S6)** (**Phage library preparation and sequencing, Methods**), enabling assessment of ARGs present in phage metagenomic data. Of 1,286 to 7,263 co-assembled contigs in the virome libraries from each sample, only 53.6% were marked with confidence as phage contigs (**Figure S6a-b**) (**Phage contig filtration, Methods**). Among these contigs, only 11.4% could be mapped to known phage genomes. Phage communities were highly individual-specific according to the Jaccard-like distances (**Figure S6c-d**).

In line with a previous report,^23^ ARGs were observed on only a few phage contigs (1 to 9 per individual with a median of 3.5, 0.17% of total phage contigs) (**Figure S7a-b, Table S4**). AsRGs were captured on five contigs from four individuals (DOX-a, DOX-b, CFX-a, and AZY-b). To identify putative host species and search for potential integration and horizontal gene transfer (HGT) events, phage contigs were mapped against metagenomic contigs to identify phage-like contigs in the metagenome (**Phage-like contig identification in metagenomes, Methods**). For each ARG-containing phage contig, up to 44 phage-like contigs (median: 1.5) were identified in the corresponding metagenomic dataset (**Figure S7c, Table S4**). Metagenomic contigs were assigned to a unique host species (**Contig binning and contig-species assignment, Methods**) by their species-specific marker genes^28^ or contig binning result.^29^ For each ARG-carrying contig, after mapping to phage-like contigs in the metagenome, up to three species were inferred as the potential host species of the contig harboring ARG (**Figure S7d**). Using short-read metagenomic assembly, 66.8% of the phage-like contigs and 75.2% phage-like contigs with ARGs were short (<1 kb). This could be a result of high variability in the isoforms of the phage-like contigs that resulted from integration into different bacterial loci and host species. As a result, 25 of 39 phage contigs with ARGs were not assigned to any host species. One phage contig carrying AsRG *tetQ* was assigned to *Bacteroides fragilis* and *B. caccae. B. caccae* was a proliferating species in individual DOX-a, suggesting that phage-mediated HGT may have been involved in the dissemination of this AsRG.

### AsRGs are frequently found on mobile genetic elements

**Figure 4.**
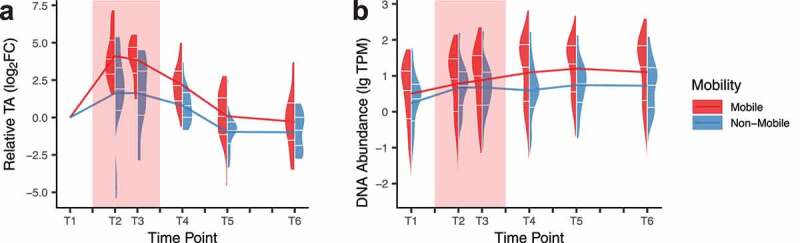
Transcriptional activity (TA) (**a**) and DNA abundance (**b**) of mobile and non-mobile AsRGs

Plasmid contigs and contigs with transposable elements were also annotated from metagenomic assemblies:^30^ 9,172 to 33,166 contigs (7.90%) were identified as potential plasmid contigs per individual (**Table S5**). We observed a strong tendency for AsRG to be present on mobile elements. In total, 39.6% of AsRGs were captured on mobile contigs compared to 15.8% for non-AsRGs (**Figure S8a**). Phage-like contigs were the majority of mobile AsRGs (66.0% compared to 11.5% for non-AsRGs) (**Figure S8b**). Also, mobile AsRGs presented higher transcriptional activities than non-mobile AsRGs (Wilcoxon rank-sum test, *p* = .03) (Figure 4a). These results suggest that mobile AsRGs were more actively involved than non-mobile AsRGs in bacterial host defense during antibiotic treatment.

FC, fold-change; lg TPM, log_10_ transcripts per kilobase per million mapped reads. The red background indicates the antibiotic administration period in all panels.

Mobile and non-mobile AsRGs did not show DNA relative abundance differentiation during treatment (Wilcoxon rank-sum test, *p =* .23); however, a discrepancy was seen after treatment (median TPM: 19.1 for mobile *vs*. 3.5 for non-mobile, Wilcoxon rank-sum test, *p =* 3 × 10^−6^) (Figure 4b). Both mobile and non-mobile AsRG abundances emerged and remained high even 3 months after the antibiotic treatment (4.3 and 2.8 folds of the baseline level in T6, respectively). The persistently high relative abundance of mobile AsRGs supported that mobile elements such as phages and plasmids contributed to the expansion of the antibiotic-specific resistance reservoir.

### Mobile AsRGs expanded the resistance reservoir via potential HGT events

**Figure 5.**
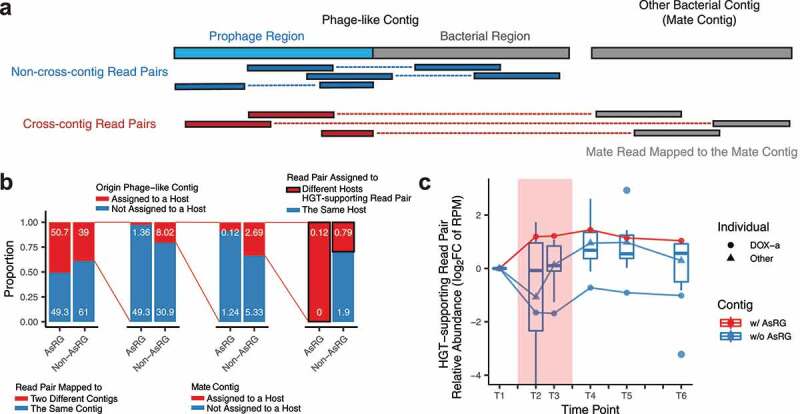
Horizontal gene transfer (HGT) potential for AsRGs mediated by bacteriophage

**Figure 6.**
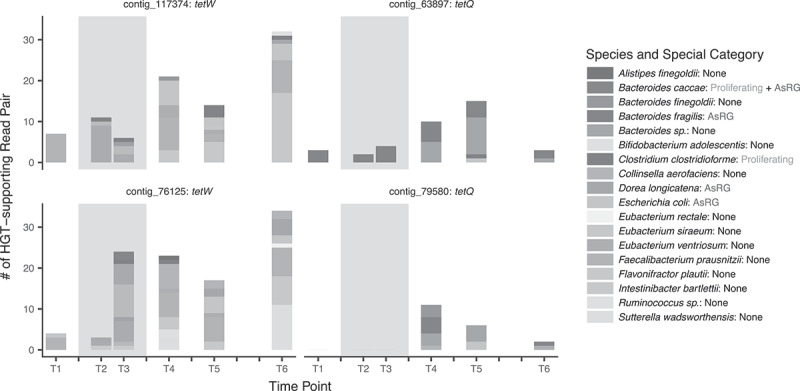
HGT-supporting read counts over time and the host species of the mate contigs

We noted that alternative phage integration isoforms could be supported by cross-contig read pairs, where a read is mapped to a prophage region, while its mate is mapped to a different contig (mate contig) (Figure 5a). To trace the ARG proliferation via potential HGT events mediated by phage integrations, these read pairs were analyzed as HGT-supporting read pairs (**HGT-supporting read pair extraction, Methods**), and the species host assigned to the mate contigs were analyzed as potential HGT-target species. As the mobility of phage-like contigs with ARGs resulted in more fragmented contig assemblies, a large number of read pairs were observed to be mapped to different contigs, especially for AsRG-carriers (50.7% *vs*. 39.0% for non-AsRG carriers) (Figure 5b). Read pairs from two contigs that mapped to different host species were analyzed as HGT-supporting read pairs, which presented in low proportion (0.12% of total mapped read pairs for AsRG-carrying contigs), while AsRG-carrying HGT-supporting read pairs were only observed in one individual treated with doxycycline (DOX-a). The relative abundance of AsRG-carrying HGT-supporting read pairs increased during treatment and remained high after treatment in DOX-a, while the non-AsRG carrying read pairs decreased and remained stable for all antibiotic-treated individuals (Figure 5c). More specifically, AsRGs *tetW* and *tetQ* from individual DOX-a exhibited a higher number of HGT-target species and HGT-supporting read pairs after treatment (Figure 6). Noticeably, several of such HGT-target species were found in proliferating species and/or AsRG carrier species.

**a**. Graphical demonstration of cross-contig read pairs. When a read was mapped to a prophage region and its mate was mapped to another contig, this read pair was analyzed as a cross-contig read pair. If the ARG-carrying contig and its mate contig were assigned to different species, the read pair was analyzed as an HGT-supporting read pair. The species assigned to the mate contig was identified as the potential HGT-target species. **b**. The proportions of ARG-carrying read pairs that are mapped to the same contig or different contigs and mapped to the same host species or different species. **c**. Number of HGT-supporting read pairs over time, summarized as reads per million mapped reads (RPM), and normalized as the log_2_ fold-change (FC) to baseline. The relative abundance of AsRG-carrying HGT-supporting read pairs increased in individual DOX-a during and after treatment, while the non-AsRG-carrying HGT-supporting read pairs decreased for DOX-a and remained stable for all individuals. The red background indicates the antibiotic administration period.

The titles for the four subpanels are the phage-like contig IDs and their carrying AsRGs. Four mate contig host species were discovered with AsRGs, and two species are proliferating species. Examples from individual DOX-a have shown that more potential HGT-target species were observed in treatment and post-treatment samples and the HGT-target species had a high probability of being a proliferating species or an AsRG carrier.

## Discussion

Antibiotics are commonly used prophylactically or to treat infections. Previous studies evaluated antibiotics as adriving factor in gut microbiome modulation and reported the resilience of dominant microbiome members after short-term antibiotic exposure.^3,6,24^ In our small cohort of antibiotic-treated healthy volunteers, we observed a rapid decline in community diversity during antibiotic administration and post-treatment, with several species becoming undetectable and not recovering within 3 months. We observed distinct responses to different antibiotics and an overall growth advantage for Gram-negative species during treatment. We note that the small size of our cohort could mean that part of the variability we observed might be attributed to differences in diet, sex, and age between individuals. We tried to account for this possibility by using randomization and by including control participants and collecting baseline samples, but certainly larger studies are needed to confirm the trends observed in this present study.

Similar to a recent study on the resistome,^3^ the overall ARG abundance did not exhibit substantial perturbations during antibiotic treatment. However, we identified expansion in AsRGs with presumed resistance to the administered antibiotics as a long-term consequence of antibiotic administration. We used metatranscriptomic sequencing to reveal the exceptionally high transcriptional activity (9-fold higher than baseline in average) of AsRGs during treatment, supporting their importance in bacterial host defense.

Reports of post-treatment expansion of some ARG families^3,24^ raised the question of how the recovery of taxonomic composition and functional profiles supported the expansion of ARGs, which must involve the persistence of ARG carrier species. As previous studies have proved the capacity of HGT event identification from paired-end signals,^31,32^ we addressed this question using paired phage library sequencing and plasmid identification, which identified significant enrichment of AsRGs within the mobilome (the collection of mobile elements in a microbiome). This would suggest an increased potential for HGT, which was supported by analysis of HGT-supporting paired sequencing reads partially mapping to the phage-like contigs. These data support the hypothesis that HGT events of AsRGs may occur during antibiotic treatment. Subsequent studies with larger cohorts could benefit from deploying longer read sequencing technology to pinpoint such HGT events.

A major limitation of this study is its reliance on sequencing data and functional annotations and computational inferences of HGT relationships. While we have deployed validated approaches for our computational assessments, our conclusions are limited by our current knowledge. We encourage further work on functional validation of antibiotic resistance genes, high-throughput cultivation, and sequencing to further increase our knowledge of antibiotic treatment on the gut microbiome.^33^

Our data and analysis suggest that the resistome of healthy individuals is broadly resilient to short-term antibiotic treatment. Yet, AsRGs stably increase in relative abundance for a period of at least 3 months until completion of antibiotic treatment, providing further evidence in support of good antibiotic stewardship.

## Materials and methods

### Study design

Ten healthy adult volunteers, aged 18–65 years, were recruited to provide stool samples over a 4-month period, before, during, and after antibiotic exposure. Participants had not received antibiotic treatment 1 year prior to study enrollment, nor they ever experienced allergic reactions to the antibiotic class used in the study. Participants did not follow special dietary habits (vegetarian or vegan). To reduce the effect of individual variation, all volunteers were treated in parallel with a single antibiotic (**Table S1**). Four antibiotics from different chemical and therapeutic classes were used: ciprofloxacin (quinolone class), cefuroxime (β-lactam class), doxycycline (tetracycline class), and azithromycin (macrolide class). As a control, stool samples from two untreated healthy individuals also were processed. Treatments were assigned through randomization by drawing an opaque envelope. After allocation, the trial was open label.

### Sampling

Six stool samples from each participant were obtained as one sample 15 days before treatment ± 1 day (T1), two samples on the third (T2) and fifth day (T3) of antibiotic treatment ± 1 day, and three samples at 15 (T4), 30 (T5), and 90 days (T6) after treatment ± 1 day. Samples were immediately stored at −18 °C at volunteers’ homes and transported to the hospital within 14 days, packed to prevent thawing. Upon arrival at the hospital, samples were stored at – 80 °C until processing.

### DNA extraction

DNA was extracted from 5 g aliquots of a frozen stool using an MO BIO PowerMax Soil DNA Extraction Kit (MO BIO Laboratories, Inc) according to the manufacturer’s protocol with a few modifications. Stool samples were stored at – 80 °C in sterile 50 ml Falcon tubes until extraction. To samples, 15 ml MO BIO PowerBead Solution and MO BIO Garnet beads were added before vortexing for 1 min at maximum speed using a horizontal vortex adopter (SI-H506, Horizontal 50 mL Tube Holder, Scientific Industries). Solution C1 (1.4 ml) was added before incubation at 65 °C for 30 min with shaking at 130 rpm. Samples were vortexed for 10 min at a maximum speed, 6 ml Solution C2 was added, and samples were incubated at 4 °C at 20 min and processed per MO BIO instructions.

### DNA purification

After extraction, DNA was purified with PowerClean Pro DNA Clean-Up Kits (MO BIO Laboratories, Inc.) according to the manufacturer’s protocol. When necessary, isolated DNA was concentrated to >50 ng/uL using a vacuum concentrator (Concentrator plus, Eppendorf). The DNA quantity was measured using Qubit 2.0 Fluorometer (Thermo Fisher Scientific Inc.). The DNA quality was measured using a NanoDrop ND-1000 spectrophotometer (Thermo Fisher Scientific) and size was examined by gel electrophoresis of 5 µl DNA on a 1% (w/v) agarose gel with RedSafe Nucleic Acid Staining Solution (iNtRON Biotechnology).

### DNA library preparation and sequencing

DNA samples were sent to Macrogen (South Korea) for library preparation and sequencing (Illumina Hiseq 2000 PE125). DNA libraries for sequencing were using TrueSeq Nano 550 bp kits (Illumina). The input template was 200 ng according to kit instructions. Sequencing depth was set as up to a minimum of 6 GB data per sample.

### RNA extraction

RNA extraction was by MO BIO PowerMicrobiome™ RNA Isolation Kit (MO BIO Laboratories, Inc.) according to manufacturer’s instructions. RNA was stored at −80 C until processing.

### RNA library preparation and sequencing

RNA samples were sent to Macrogen (South Korea) for library preparation and sequencing (Illumina Hiseq 2000 PE125). For each sample, 2.5 μg total RNA was used as input for rRNA, which was processed using Ribo-Zero Gold rRNA removal kits – Epidemiology (Illumina). RNA libraries were constructed using Trueseq RNA library preparation kits (Illumina) according to manufacturer’s instructions. Two platforms were used for RNA sequencing with balanced data output (Illumina Hiseq 2500 PE125 and Illumina NextSeq PE75). Sequencing depth was set as up to a minimum of 2 GB per sample.

### Phage DNA extraction

Phage particles were isolated from 5 g aliquots of frozen stool with 50 ml Phage Buffer (10 mM Tris, pH 7.5, 10 mM MgCl2, 68 mM NaCl, 1 mM CaCl2) before homogenization by vortexing for 20 min at the highest speed (SI-H506, Horizontal 50-mL Tube Holder, Scientific Industries). Samples were centrifuged 3 times at 4 °C: 2 min at 872 × *g*, 10 min at 3800 × *g*, and 20 min at 7500 × *g*. After each centrifugation, supernatants were transferred to new 50 ml Falcon tubes and pellets discarded. Supernatants were filtered through 0.22 µm filters (EMD Millipore Sterivex-GP SVGPL10RC Polyethersulfone Filter Unit, Millipore). To concentrate virus particles, 10 ml of filtered supernatants were concentrated to 1 ml by centrifugation with 100 Da Amicon Ultra filters (Amicon Ultra-15 Centrifugal Filter Units, Millipore) at 3488 × *g* at 15 °C. Supernatants in Amicon tubes were washed two times with 5 ml Phage Buffer and volumes adjusted to 1 ml. Supernatants were filtered through 0.45 µm syringe filters (Cellulose acetate membrane syringe filter, Filter Technology) into 1.5 ml phase-lock gel tubes (5 PRIME), 40 µL lysozyme (10 mg/mL, Sigma-Aldrich) was added, and filtrates incubated for 30 min at 37 °C under shaking at 300 rpm. After the incubation, 400 µL chloroform was added to samples before incubating for 15 min at room temperature with gentle inversion every 2 minutes. Samples were centrifuged at 14,000 × *g* for 5 min at room temperature and supernatants transferred to 1.5 ml Eppendorf tubes. A mix of 500 U bovine pancreas DNase I recombinant (Roche), 33 U Baseline-ZERO DNase (Epicenter), 6 U Salt Active Nuclease (ArcticZymes), and 500 U RNase A (Roche) was added to samples with 100 µl 10× Incubation buffer (Roche) for incubation at 37 °C for 90 min followed by DNase inactivation at 75 °C for 10 min. After the DNase/RNase treatment, phage particles were stored overnight at 4 °C. Phage DNA was extracted using Phage DNA Isolation Kits (Norgen Biotek) according to the manufacturer’s protocol. DNA quantity was measured using a Qubit 2.0 Fluorometer (Thermo Fisher Scientific Inc.). Phage DNA samples were stored at −80 °C.

### Control for bacterial contamination in phage DNA extractions

Full-length 16S rRNA gene (1503 bp) was amplified with 16S_up (AAGAGTTTGATCCTGGCTCAG) and 16S_lp (TACGGCTACCTTGTTACGACTT) primers^34^ from *Pseudomonas aeruginosa* PAO1 reference strain (NC_002516) by quantitative PCR (qPCR). The template for qPCR reactions with SYBR Green Master Mix (Thermofisher) was 0.5 ng phage DNA. All samples were amplified by qPCR with triplicates of standards with known gene copy numbers and negative controls. Standards were 10-fold dilutions of purified, full-length 16S rRNA gene amplicons. Phage DNA samples with higher cycle amplifications (above 28 Ct corresponding 10^2^ gene copies) were discarded and phage DNA extraction was repeated. Phage DNA below the threshold of detection for 16S rRNA gene copies (10^2^) was used for next-generation sequencing library preparations.

### Phage library preparation and sequencing

Phage DNA libraries were prepared using KAPA HyperPlus Kits (Kapa Biosystems). All steps were on ice except two clean-ups that were at room temperature. Samples of 2.5 ng phage DNA were diluted in 17.5 µl 10 mM Tris-HCl (pH 8.0–8.5). Enzymatic fragmentation was achieved by adding 2.5 µl KAPA Frag Buffer (10X) and 5 µl of KAPA Frag Enzyme to DNA dilutions in PCR tubes. Tubes were vortexed gently and spun down briefly, then incubated at 37 °C for 30 min in a thermocycler that was pre-cooled to 4 °C. After adding End-repair and A-tailing buffer, PCR tubes were vortexed and spun down and immediately incubated at 65 °C for 30 min in a thermocycler with the lid preheated to 85 °C. Adapter ligation reactions were in the same tubes with an addition of 3.75 µl PCR-grade water, 15 µl Ligation Buffer, 5 µl DNA ligase, and 1.25 µl 750 pM single adapters (Pentabase). Tubes were mixed thoroughly and centrifuged briefly and incubated at 20 °C for 60 min. Products were cleaned using Agencourt AMPure XP reagent at a ratio of 1:0.7 (adapter ligation reaction product: reagent). Reagent and product were mixed by pipetting 10 times followed by short spin down centrifugation. Mixtures were left for 15 min at room temperature to bind DNA to beads. Beads were captured by magnets for 5 min and supernatants were carefully removed and discarded. With tubes still on the magnet, 200 µl freshly prepared 80% ethanol was added with incubation for 30 s. Ethanol was discarded and the ethanol washes repeated. Tubes were left on the magnet for 5 min to dry the beads before resuspending them in 12.5 µL 10 mM Tris-HCl, pH 8.0–8.5 and vortexing for 30 s. Beads were incubated at room temperature for 2 min to elute DNA, then captured by a magnet for 5 min. Supernatants were transferred to new tubes and 12.5 µl 2X KAPA HiFi HotStart ReadyMix and 2.5 10X KAPA Library Amplification Primer Mix were added to 10 µl adapter-ligated library. Reagents were mixed and tubes centrifuged briefly. Library amplification used the cycling protocol: 1 cycle 98 °C, 45 s; followed by 14 cycles of 98 °C for 15 s, 60 °C for 30 s, and 72 °C for 30 s, with a final extension at 72 °C for 1 min. Post-amplification cleanup was as described above. Concentrations were measured using a Qubit 2.0 Fluorometer (Thermo Fisher Scientific Inc.), and library size (average 500–900 bp) was determined using a Bioanalyzer (Agilent 2100 Bioanalyzer system, Agilent Technologies). Libraries are pooled and sequenced on a MiSeq platform (PE300).

### Nucleic acid extraction and sequencing control

Reference strain *E. coli* MG1655 transformed with pzZE21mCherry was used as a control for the nucleic acid extraction and sequencing protocols. Sequencing reads were mapped to a reference genome using CLC Genomic Workbench (version released in 2015). More than 99.5% of reads are mapped back to the reference genome.

### Sequencing data quality control

All raw reads from DNA, RNA, and phage libraries underwent quality trimming using a previously described pipeline^35^ to filter out adapters and universal primer sequences, low-quality bases (<Q20), reads shorter than 75 bp PE125 (and 30 bp for PE75 read obtained on NextSeq), duplication reads and reads mapping to the human genome with over 95% identity. Computational scripts are at https://github.com/TingtZHENG/VirMiner/. Quality control results are summarized in **Data S1.**

### rRNA removal from RNA sequencing clean data

Removal of rRNA was by *riboPicker* (version 0.4.3)^36^ against the non-redundant rRNA database (*riboPicker*, downloaded from http://edwards.sdsu.edu/ribopicker/rrnadb/rnadb_2012-01-17.tar.gz) with arguments “-c 80 -i 90”. The results are summarized in **Data S1**.

### In silico estimation of bacterial contamination in virome

We compared the 16S rRNA gene content in virome and whole metagenome samples. First, viral reads were truncated to 125 bp to match the read length of the whole metagenome dataset. Subsequently, both datasets were mapped against the SILVA database (v.123)^37^ using *bwa mem* v. 0.7.15^38^ and the number of unique reads mapped with at least 90% identity was counted for both. Finally, the percentage of 16S reads within the entire read set of each sample was calculated and compared with published virome datasets (MetaVir)^39^ (**Figure S8**).

### De novo assembly

The *de Bruijn* graph-based assembler *IDBA-UD* (v. 1.1.1)^40^ was used for *de novo* assembly. Clean reads from all time points for each individual were pooled for the co-assembly of metagenome and virome, respectively. For metagenomes, parameters for *IDBA-UD* were: “-mink 40 -maxk 100 -step 10 -num_threads 24 – min_contig 300 -pre_correction”. For viromes with PE300 sequencing, *k* = 180 was selected as the max *kmer* length. Two modifications were made in the source code before compiling *IDBA*_*UD*: in file src/basic/kmer.h, constant *kNumUint64* was changed from 4 to 8 to allow maximum *kmer* length beyond 124; in file src/sequence/short_sequence.h, constant kMaxShortSequence was set to 512 to support longer read length. Virome co-assembly used parameters: “-mink 20 -maxk 180 -step 20 -num_threads 24 – min_contig 800 -pre_correction”. After co-assembly, paired-end reads from the metagenomic DNA/RNA libraries were aligned to the metagenomic assemblies, while the viral DNA reads were aligned to the viromic assemblies using *bwa* “*mem*” model (v. 0.7.15).^38^ Statistics of mapped and unmapped reads were calculated using *samtools* with function “*flagstat*” (v. 1.3.1).^41^ Overall, final assemblies had mean mapping percentages of 82.3% to 91.4% for metagenomic contigs (**Table S6**) and 76.4% to 93.3% for viromic contigs (**Table S7**). *Samtools* functions *“depth -aa”* and *“idxstats”* were used to calculate contig coverage and depth and per-locus depth.

#### Updated phage orthologous group (uPOG) database

We used an updated POG database for phage gene annotation – uPOG.^42^ The uPOGs are available on our website (http://147.8.185.62/VirMiner/downloads/updated_POG_database/).

### Open reading frame (ORF) prediction and annotation

For metagenomes, *MetaGeneMark* was adopted to predict coding DNA sequence (CDS) regions in assembled metagenome contigs using default parameters.^43^ The functional COG category for each protein was assigned using the National Center for Biotechnology Information *rps-BLAST*^44^ with the parameter “-e 1e-5”. Protein sequences were aligned to the Kyoto Encyclopedia of Genes and Genomes (KEGG) database^45^ using *Diamond blastp*^46^ with the parameter “-e 1e-5”. For viromes, ORFs were predicted by *GeneMarkS* v4.3^47^ with the parameter “–phage”. Predicted ORFs from metagenomic and viromic contigs were mapped against Pfam,^48^ POG 2012,^49^ and uPOG databases by *DIAMOND blastp*^46^ with parameters “–id 70 -e 1e-5” and the top hits were selected.

### Antibiotic resistance gene homolog (ARG) annotation

The CARD database^50^ and the accompanying *Resistance Gene Identifier (RGI)* pipeline were used to annotate ARGs in the metagenome and the virome. For metagenomes, protein sequences of predicted ORFs were used as input. For viromes, input was viral contig sequences. The *RGI* hits with “Perfect” and “Strict” identification were used as qualified ARG annotations.

### Antibiotic-specific resistance gene homolog (AsRG) annotation

To identify ARG homologs with proven resistance to each antibiotic given to the study participants, we compiled a list of AsRGs from previous literature (**Table S3**). Genes with no annotation hit in the metagenomes were removed. The list was further verified by CARD^50^ and only genes listed as “confers_resistance_to_drug” in “Sub-Term(s)” of each antibiotic were kept. For potential AsRGs in the mobilome (virome, phage-like contigs, plasmid contigs, and contigs with TE), only the *RGI* hits with “Perfect” or “Strict” identification and a minimum identity of 95% were kept.

### Calculation of transcriptional activity

A gene or contig’s DNA and RNA abundances were calculated by transcripts per million mapped reads (TPM). Only genes with detectable abundances (TPM > 1e-5) across all time points were analyzed for DNA abundance. For the genes, contig, or gene sets with detectable DNA and RNA abundances (TPM > 1e-5) across all time points, transcriptional activity (TA) was calculated as TA = RNA (TPM)/DNA (TPM). Log_2_ transformation for TA was applied before statistical tests.

### Taxonomic assignment of phage contigs

The RefSeq^51^ database of viral reference genomes Release 81 (March 2017) was used to map contigs from each assembly using *blastn*^44^ with filtration criteria: *E* < 1e-4, identity > 70% and coverage > 50%. Contigs that were shorter than 3 kb were discarded. The relative abundance of each contig with a reference viral genome hit was calculated as transcripts per kilobase per million mapped reads (TPM). For each viral family, the relative abundance was calculated as the sum of TPM of all contigs assigned to the viral family.

### Phage contig verification by VirSorter and VirFinder

All phage library contigs were analyzed by *VirSorter*^52^ with “virome database” and “virome de-contamination” modes. Contigs classified in any viral categories or as prophages were considered viral. *VirFinder*^53^ analysis used default settings and contigs with a false discovery rate (*FDR*) below 0.05 were considered viral.

### Phage-like contig identification in metagenomes

Phage library contigs were mapped against metagenomic contigs to find target phage-like contigs and phage integration sites in metagenomes. *MegaBlast*^44^ was applied with parameters “–id 90 -e 1e-5”. Mapped regions with unmapped gaps smaller than 100 bp were catenated. Metagenomic contigs with coverage greater than 50% of the phage contig were identified as phage-like contigs in metagenomes.

### Phage contig filtration

Phage library contigs meeting at least two of the following criteria were marked as confident phage contigs: 1) annotated with uPOG gene; 2) annotated with viral genes from PFam; 3) mapped to known phage genome in RefSeq; 4) identified as viral contig by *VirSorter*; 5) identified as viral contig by *VirFinder*; 6) mapped to at least three target phage-like contigs in the metagenome. Only confident phage contigs were used in downstream analyses. Contigs meeting only one criterion were marked as “suspicious phage contigs” with others marked as “contaminant contigs.

### Plasmid contig annotation

Due to the complexity of a metagenome and the presence of homologous sequences from different species, metagenomic assemblies always yield fragmented contigs and invisible mis-assemblies. Thus, a traditional plasmid identification tool that relies on circular contig assembly of high quality may not be the best practice for metagenome assemblies. Thus, we employed *PlasFlow*,^30^ a neural network-based tool to identify potential plasmid contigs from models trained by *kmer* frequencies. Among the metagenomic contigs longer than 1kbp, plasmid contigs were annotated with *PlasFlow* v1.0 with the parameters and default models (*k* = 5, 6, 7, respectively). Among the results, entries marked as “unclassified” or “chromosome” were discarded, and contigs binned as “plasmid” with a probability score over 0.7 were kept as plasmid contigs. The ARGs on these plasmid contigs were marked as mobile ARGs.

### Transposable element annotation

These elements in metagenomic contigs were annotated using the database *ISFinder*.^54^ ORF protein sequences and contig nucleotide sequences were mapped against, respectively, *ISFinder* protein and nucleotide databases using *Diamond blastp*^46^ or *blastn*^44^ with parameters “–id 70 -e 1e-5”. Contigs with mapped transposable elements (TE) were marked as contigs with transposable elements.

### Identification of mobile contigs and mobile AsRGs

Metagenomic contigs annotated as phage-like contigs, plasmid contigs, or contigs with transposable elements were classified as mobile contigs. Identified AsRGs on these contigs were classified as mobile AsRGs. For each individual and each species ARG profile, identified AsRGs with no mobile copies in that species were classified as non-mobile AsRGs.

### Species-specific marker gene annotation

Species marker gene profiles were obtained through *MiDAS*^28^ with arguments “run_midas.py genes -s very-sensitive – species_cov 0.1”, for samples with efficient read depth (“merge_midas.py genes -sample_depth 0.1”). Functional profiles (Gene Ontology, KEGG Orthology, Enzyme Commission number (EC)) were further summarized based on annotations from the MiDAS reference database (midas_db_v1.2). Metagenomic ORFs were mapped against this marker gene database using *Diamond blastp*.^46^ All hits with identity over 70% and *E*-value less than 1e-5 were kept for contig-to-species assignment.

### Contig binning and contig-species assignment

For each individual, co-assembled metagenomic contigs were binned using *MaxBin* 2.2.4^29^ with the default parameters. For each contig or contig bin, species-specific marker genes were summarized. If more than 70% of the species-specific marker genes appeared in a contig or a contig bin could be assigned to a single candidate species with only one candidate species identified, then the contig or contig bin was assigned to that species. If a contig was successfully assigned to a host species, the species for its contig bin was then ignored. If a contig could not be assigned to a host species, while its bin could be assigned to a host species, then the contig bin’s host species was assigned to the contig. All the functional genes on a contig, including ARGs, were assigned to the contig’s host species.

### HGT-supporting read pair extraction

Reads mapped to phage-contig homologous regions on phage-like contigs were extracted by *samtools* function “*view*”^41^ and mate reads were extracted from bam files using grep command. Read pairs with reads mapped to different contigs were kept for cross-contig read-pair analysis. A read pair is defined as an HGT-supporting read pair if the ARG-carrying contig and its mate contigs were assigned to different host species.

### Metagenome taxonomic assignment

The relative abundance of taxa was analyzed using *MetaPhlAn2* with default settings.^55^ Different databases may use different names for the same species. Thus, we aligned the species names used in this manuscript, *MetaPhlAn2, and MiDAS* (used for the contig-species assignment) and summarized the information in **Data S2**.

### Community diversity and dissimilarity

For metagenomes, species-level taxonomic profiles were used as input for alpha-diversity and beta-diversity analyses. Alpha-diversity was represented by Shannon index and beta-diversity by Bray–Curtis distances, both using the *vegan R* package.^56^ For viromes, alpha-diversity (Shannon index using *vegan*) was calculated on the relative abundance matrix of confident phage contigs (in TPM). For beta-diversity, *MASH*^57^ *MinHash* sketch strategy for estimating the Jaccard index was used to estimate dissimilarity between samples. Briefly, a mash sketch for each read file from each time point was derived and distances of all-against-all sketches were calculated. Ordinations for the beta-diversity analysis were calculated by nonmetric multidimensional scaling for illustrations.

### Categorization of special species

Species were categorized into three groups based on the following criteria: 1. Disappeared: the species undetected in all post-treatment samples (T4 to T6); 2. New: the species undetected in the baseline, but persistently abundant (relative abundance > 0.1%) in all post-treatment samples (T4 to T6); 3. Proliferating: the species meeting any of the two criteria: 1) a species with a relative abundance < 0.1% in baseline and reached 5% in T2 or T3, or 2) a species with a relative abundance ≥ 0.1% in baseline and increased by at least 10-fold in the relative abundance in T2 or T3.

### Growth rate index (GRiD) calculation

GRiD was calculated according to a previously published method^25^ with the following parameters: “grid multiplex -d/sbidata/shared2/GRiD/Stool -*p* -c 0.2 -m”. GRiD may generate not applicable (NA) values for all species in one sample; thus, such time points (T3 for DOX-a and T4 for CTR-a) were considered invalid and excluded from analyses. For statistics, we kept only the species with no NA values across all valid time points.

### Gram-positive and gram-negative species

Bacterial species and strain information were acquired from PATRIC,^26^ visited in November 2017. Bacterial species with all strains with the same (or missing) Gram-staining type were assigned as Gram-positive or Gram-negative species. Species with conflicting Gram-staining types for different strains were categorized as dubious.

### Statistics and data visualization

Statistics were done in *R,^58^* with data visualization by *R* and corresponding packages including *ggplot2*,^59^ *grid,^60^ gridExtra,^61^ RColorBrewer,^62^ ellipse,^63^* and *pheatmap*.^64^ Two-tailed Wilcoxon signed-rank test was performed for comparisons of paired data in nonparametric statistics. Two-tailed Wilcoxon rank-sum tests were performed for comparisons of unpaired data in nonparametric statistics. Adonis tests were applied to address community dissimilarity. Fisher’s exact tests (only when there are less than 5 observations in a table cell) or Pearson’s chi-square tests were performed for 2 × 2 tables for independence test. The significance threshold was set to *p* < .05 or false discovery rate (*FDR*) < 0.05.

## Supplementary Material

Supplemental Material

## Data Availability

Raw sequencing data is published at NCBI SAR with the project ID PRJNA588313 and sample category ID SAMN13241759. Raw data for DNA libraries: SRR10423895 to SRR10423894; RNA libraries: SRR10420935 to SRR10420934; virome libraries: SRR10417995 to SRR10418053. Intermediate data and codes are available at http://sbb.hku.hk/Resistome.
