# Supplementary material for: Expansion and persistence of antibiotic-specific resistance genes following antibiotic treatment
Source: Gut Microbes. 2021 Mar 28;13(1):1900995. doi: 10.1080/19490976.2021.1900995 (PMC8018486; doi:10.1080/19490976.2021.1900995)
Supplement: Supplemental Material [file KGMI_A_1900995_SM9325.docx]

**Supplemental Figures**

**Figure S1. The study design**. T, timepoint. T1 is the baseline, T2 and T3 are the time points during antibiotic treatments. T4 to T6 are post-treatment. Ten individuals were employed for four different antibiotic treatments and one control group.

**Figure S2. The taxonomic profiles of the metagenomic samples. (a)** The taxonomic profile at the phylum level. AZY, azithromycin; DOX, doxycycline; CFX, cefuroxime; CIP, ciprofloxacin; CTR, control; T, timepoint. Baseline: T1, day -15 before treatment. During treatment: T2, day 3 and T3, day 5. After treatment: T4, day 15; T5, day 30 and T6, day 90. Taxonomic profiles were constructed using MetaPhlAn2^1^. In the majority of samples, Bacteroides and Firmicutes were dominant at the phylum level. The during-treatment samples from individual CIP-b were dominated by Proteobacteria. **(b)** The relative abundances of all proliferating species (Proliferating) and species harbouring antibiotic-specific resistance gene homologous (AsRGs). Species relative abundances were normalized to *Z* scores across all samples before visualization. Row-side colour bars indicate a species’ Gram-staining type and if a species is a proliferating species or an AsRG carrier. Column-side colour bars indicate the treatment period and the antibiotic administered to the individual.

**
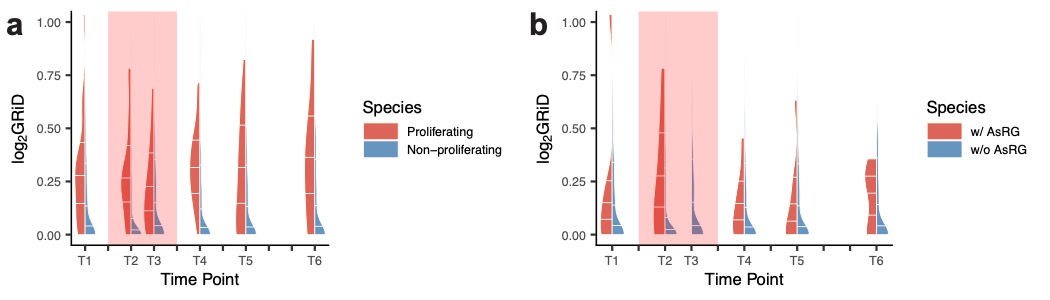
**

**Figure S3. The growth rate indices (GRiD) for different species categories.** GRiDs were calculated following a previously published method^2^. In both panels, the split violin plots demonstrate the GRiD distribution, with the 25%, 50%, and 75% quantiles marked with white ticks. The line graphs represent the mean values. **(a)** The GRiDs of the proliferating species during treatment (Proliferating) and the non-proliferating species. The proliferating species had significantly higher growth rates regardless of the treatment period (median of GRiD: proliferating 1.2, non-proliferating 1.0, Wilcoxon rank-sum test, *p* = 2 × 10^-10^). **(b)** The growth rates of antibiotic-specific resistance gene homologous (AsRG) carriers (median of GRiD: 1.1) and the species harbouring no AsRGs (median of GRiD: 1.0), with no statistical significance achieved in all time points (Wilcoxon rank-sum test, *p* > 0.05).

**Figure S4. Statistics of antibiotic-specific resistance gene homologous (AsRGs) in metagenomic contigs.** **(a)** The AsRG copy number in the metagenomic contigs. In the 8 antibiotic-treated individuals, 17 AsRGs were identified: 9 genes (all from *tet* family) for doxycycline (DOX), 3 (all from *erm* family) for azithromycin (AZY), 4 for cefuroxime (CFX), and 1 for ciprofloxacin (CIP). Among these genes, AsRGs for doxycycline had the highest gene copy numbers (36 and 50 for individuals DOX-a and DOX-b) in metagenomic contigs. **(b)** The number of host species for each AsRG**.** The *tet* family had the broadest bacterial hosts, followed by *cep* and *erm*. AsRGs for doxycycline had the widest distribution among host species (8 and 15 AsRG-host pairs, 6 and 8 unique host species for DOX-a and DOX-b respectively).

**Figure S5. Antibiotic-specific resistance gene homologs (AsRG) profiles in the metagenome. (a)** DNA abundance. AsRGs’ DNA abundances were calculated by transcripts per million mapped reads (TPM) and normalized to *Z* scores among all samples before visualization. **(b)** Transcriptional activity (TA). AsRGs’ transcriptional activities were calculated by RNA (TPM) / DNA (TPM) and normalized to *Z* scores among all samples before visualization. Row-side color bars indicate if an ARG is an AsRG in different treatments. Column-side colour bars indicate the treatment period and used antibiotics of a sample.


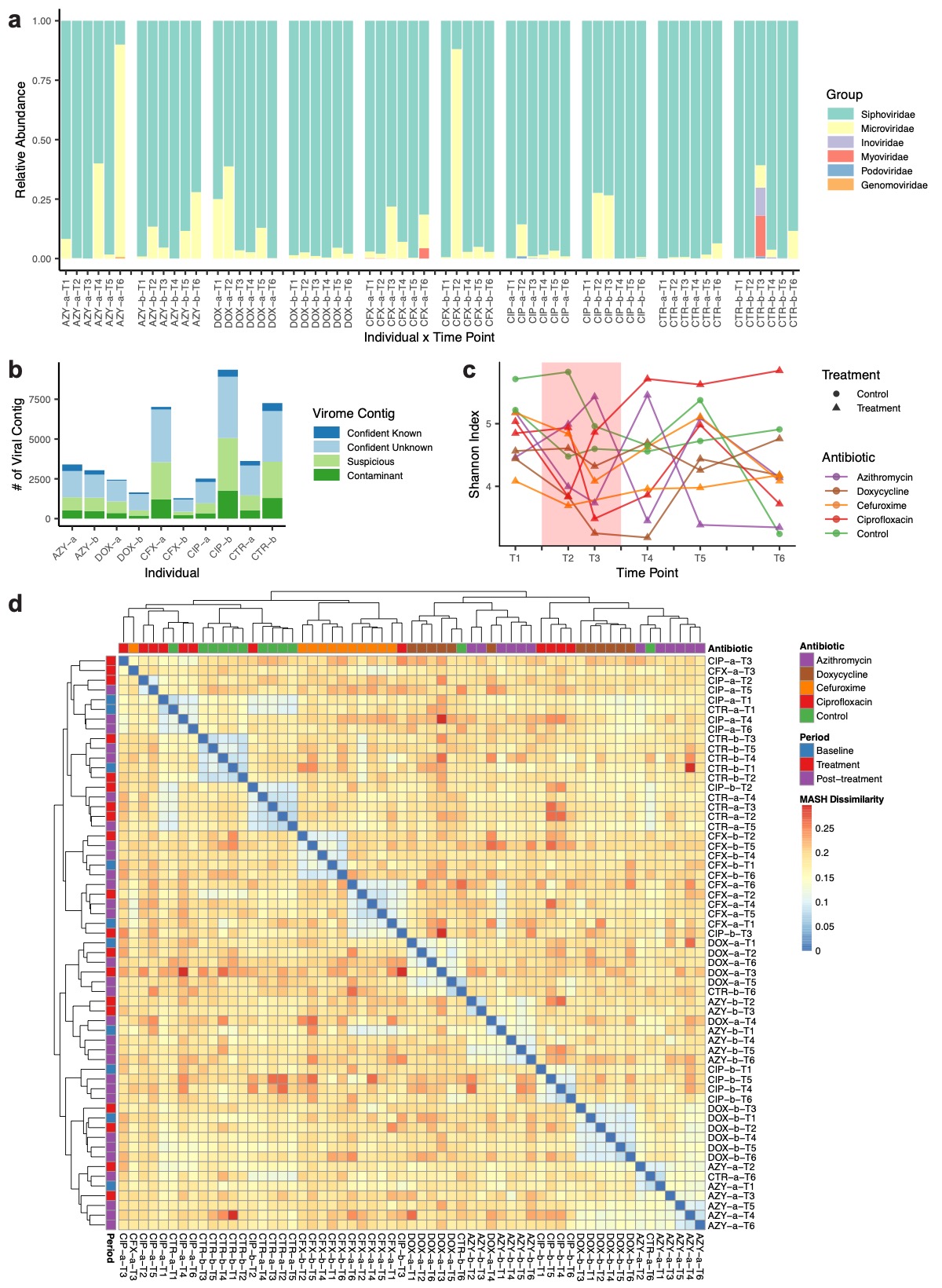


**Figure S6. The virome profile. (a)** Statistics for confident and dubious phage contigs. AZY, azithromycin; DOX, doxycycline; CFX, cefuroxime; CIP, ciprofloxacin; CTR, control. Phage library contigs meeting at least two of the following criteria were marked as confident phage contigs: 1) annotated with uPOG gene; 2) annotated with viral genes from PFam; 3) mapped to known phage genome in RefSeq; 4) identified as viral contig by VirSorter; 5) identified as viral contig by VirFinder; 6) mapped to at least 3 target phage-like contigs in the metagenome. Contigs met only one criterium were marked as dubious, while contigs met no criterium were marked as contaminants. Of 1,286 to 7,263 co-assembled contigs in the virome libraries from each sample, only 53.6% were marked with confidence as phage contigs. **(b)** The Phage taxonomic profiles. Phage contigs were mapped against known phage genomes in RefSeq (release 81, March 2017) using blastn (*E* < 1e-4, identity > 70% and coverage > 50%). Contigs that shorter than 3 kb were discarded. The abundance of each contig with a reference viral genome hit was calculated as transcripts per kilobase per million mapped reads (TPM). For each viral family, relative abundance was calculated as the sum of TPM of all contigs assigned to the viral family. Siphoviridae was identified as the most abundant phage group. **(c)** Phage library alpha-diversity in the Shannon index. Shannon index was calculated on the abundance matrix of confident phage contigs (in TPM). The Shannon indices for different antibiotics were shown. Phage communities’ alpha diversities declined during antibiotic treatment. **(d)** The dissimilarity of phage compositions. MASH^3^ MinHash sketch strategy for estimating the Jaccard index was used to estimate dissimilarity between samples. Briefly, a mash sketch for each read file from each time point was derived and distances of all-against-all sketches were calculated. Ordinations for beta-diversity analysis were calculated by nonmetric multidimensional scaling for illustrations. Color bars indicate sample information including treatment period and antibiotic type. Phage communities were highly individual-specific.

**Figure S7. Antibiotic-specific resistance gene homologous (AsRGs) profile in the virome. (a)** AsRG presence on phage contigs. AZY, azithromycin; DOX, doxycycline; CFX, cefuroxime; CIP, ciprofloxacin; CTR, control. AsRGs were discovered in phage contigs in four individuals (AZY-b, CFX-a, DOX-a, and DOX-b). **(b)** The number of metagenomic contigs that aligned to phage contigs with AsRGs. Phage-like contigs in metagenomes were identified with MegaBlast^4^ with parameters “--id 90 -e 1e-5”. Phage-like contigs with AsRGs were identified in four individuals. **(c)** The number of host species that the phage-like contigs in metagenomes could be assigned to. For each ARG in the virome, up to three species were inferred as the potential host species of the contig harbouring ARG.

**Figure S8.** **The** **mobility of the metagenomic contigs carrying antibiotic-specific resistance gene homologous (AsRGs) and non-antibiotic-specific resistance gene homologous (non-AsRGs).** **(a)** The numbers and proportions of ARG-carrying contigs. AsRGs showed a greater tendency to appear on mobile genetic elements. 39.6% of AsRGs were captured on mobile contigs compared to 15.8% for non-AsRGs. **(b)** AsRGs on different mobile elements: phage-like contigs, plasmids, and other contigs with transposable elements (TE). Phage-like contigs were the majority of mobile AsRGs (66.0% compared to 11.5% for non-AsRGs).

**Figure S9. Estimated bacterial presence in viromes.** Gene content for 16S rRNA was compared between paired whole metagenome-virome samples and published virome data sets (MetaVir)^5^. The red horizontal line corresponds to the 0.02% 16S rRNA threshold proposed by Roux et al^6^. AZY, azithromycin; DOX, doxycycline; CFX, cefuroxime; CIP, ciprofloxacin; CTR, control. Overall, the bacterial contaminants were at a low level. Only three samples from individual CIP-b have significant contaminants (> 0.02%).

**Supplemental Tables**

**Table S1. Study design.**

| **Individual ID** | **Antibiotic** | **Abbreviation** | **Dose** | **Administration** | **Treatment duration / day** | **Timepoint available** | **Age / year** | **Sex** |
| --- | --- | --- | --- | --- | --- | --- | --- | --- |
| **AZY-a*** | Azithromycin | AZY | 500 mg day 1 / 250 mg day 2-5 | Oral, once a day | 5 | T1 – T6** | 23 | F |
| **AZY-b** | Azithromycin | AZY | 500 mg day 1 / 250 mg day 2-5 | Oral, once a day | 5 | T1 – T6 | 25 | M |
| **DOX-a** | Doxycycline | DOX | 100 mg | Oral, twice a day | 7 | T1 – T6 | 44 | M |
| **DOX-b** | Doxycycline | DOX | 100 mg | Oral, twice a day | 7 | T1 – T6 | 35 | F |
| **CFX-a** | Cefuroxime | CFX | 500 mg | Oral, twice a day | 5 | T1 – T6 | 51 | F |
| **CFX-b** | Cefuroxime | CFX | 500 mg | Oral, twice a day | 5 | T1, T2,  T4 – T6 | 56 | F |
| **CIP-a** | Ciprofloxacin | CIP | 500 mg | Oral, twice a day | 5 | T1 – T6 | 30 | F |
| **CIP-b** | Ciprofloxacin | CIP | 500 mg | Oral, twice a day | 5 | T1 – T6 | 29 | F |
| **CTR-a** | Control | CTR | - | - | - | T1 – T6 | 41 | M |
| **CTR-b** | Control | CTR | - | - | - | T1 – T6 | 45 | M |

* AZY, azithromycin; DOX, doxycycline; CFX, cefuroxime; CIP, ciprofloxacin; CTR, control.

** T, timepoints. Baseline: T1, day -15 before treatment. During treatment: T2, day 3 and T3, day 5 in treatment. After treatment: T4, day 15, T5 day 30 and T6 day 90.

**Table S2. The proliferating species, disappeared species, and AsRGs carriers.**

| **Individual** | **Proliferating Species** | **Disappeared Species** | **Species w/ AsRG**** |
| --- | --- | --- | --- |
| **AZY-a*** | *Bacteroides eggerthii, Ruminococcus torques, Bacteroides coprocola, Lactococcus* phage jm2 | *Parabacteroides* unclassified | *Bacteroides xylanisolvens* |
| **AZY-b** | *Eubacterium rectale, Paraprevotella clara, Paraprevotella* unclassified*, Clostridium sp.* ATCC BAA*, Ruminococcus sp.* | *Bifidobacterium adolescentis, Bifidobacterium longum, Collinsella aerofaciens, Bacteroides coprocola, Bacteroides eggerthii, Bacteroides stercoris, Bacteroidales bacterium ph8, Parabacteroides johnsonii, Alistipes onderdonkii, Eubacterium eligens, Coprococcus sp.* ART55*, Ruminococcus bromii, Ruminococcus lactaris, Ruminococcus sp., Mitsuokella multacida, Veillonella* unclassified*, Sutterella wadsworthensis, Bilophila* unclassified*, Akkermansia muciniphila* | *Enterococcus faecium*, *Clostridium sp. ATCC BAA*, *Blautia obeum**** |
| **DOX-a** | *Roseburia intestinalis, Bacteroides caccae, Paraprevotella clara, Paraprevotella* unclassified*, Clostridium clostridioforme* | *Prevotella copri, Clostridium sp.* ATCC BAA*, Eubacterium hallii, Clostridium bartlettii, Subdoligranulum sp., Escherichia coli, Akkermansia muciniphila* | *Clostridium bolteae*, *Bacteroides ovatus, Escherichia coli, Dorea longicatena, Bacteroides caccae, Bacteroides fragilis* |
| **DOX-b** | *Bacteroides fragilis, Bacteroides thetaiotaomicron* | *Bifidobacterium adolescentis, Bacteroides clarus, Ruminococcus sp., Streptococcus* phage ALQ13*, Streptococcus* phage Abc2 | *Bacteroides fragilis, Clostridium clostridioforme, Parabacteroides distasonis, Anaerotruncus colihominis, Flavonifractor plautii, Faecalibacterium prausnitzii, Eubacterium eligens, Blautia sp. KLE****, *Coprobacillus sp.* *** |
| **CFX-a** | *Bacteroides caccae, Bacteroides cellulosilyticus, Bacteroides ovatus, Bacteroides thetaiotaomicron, Bacteroides xylanisolvens, Barnesiella intestinihominis* | *Ruminococcus bromii, Dialister invisus* | *Bacteroides thetaiotaomicron, Bacteroides ovatus* |
| **CFX-b** | *Clostridium clostridioforme, Clostridium bolteae, Eubacterium siraeum, Roseburia intestinalis* | *Eubacterium rectale, Roseburia inulinivorans* | *Bacteroides ovatus, Bacteroides caccae, Bacteroides fragilis, Escherichia coli* |
| **CIP-a** | *Bacteroides finegoldii, Bacteroides caccae, Roseburia hominis* |  |  |
| **CIP-b** | *Eubacterium ramulus, Escherichia coli* | *Bifidobacterium adolescentis, Bifidobacterium longum, Collinsella aerofaciens, Bacteroides thetaiotaomicron, Bacteroidales bacterium ph8, Barnesiella intestinihominis, Odoribacter splanchnicus, Parabacteroides distasonis, Parabacteroides goldsteinii, Parabacteroides johnsonii, Parabacteroides merdae, Alistipes finegoldii, Alistipes putredinis, Alistipes shahii, Clostridium sp.* L2*, Eubacterium eligens, Coprococcus eutactus, Dorea longicatena, Sutterella wadsworthensis, Bilophila* unclassified*, Bacteroides* phage B124 | *Escherichia coli* |
| **CTR-a** | *Bacteroides massiliensis* | *Lactococcus* phage P680*, Lactococcus* phage jm2*, Lactococcus* phage phi7*, Leuconostoc* phage phiLN04*, Ageratum* yellow vein Singapore alphasatellite |  |
| **CTR-b** | *Alistipes finegoldii, Alistipes onderdonkii, Eubacterium siraeum, Odoribacter splanchnicus, Paraprevotella clara* | *Parabacteroides johnsonii, Parabacteroides* unclassified*, Paraprevotella clara, Catenibacterium mitsuokai, Mitsuokella multacida* |  |

* AZY, azithromycin; DOX, doxycycline; CFX, cefuroxime; CIP, ciprofloxacin; CTR, control.

** AsRG, antibiotic-specific resistance gene homologous.

*** Species with assigned bins or contigs but missing in the relative abundance profiles.

**Table S3. Antibiotic-specific resistance gene *.**

| **Antibiotic** | **Antibiotic-specific resistance gene** | **References** |
| --- | --- | --- |
| **Azithromycin** | *ermB* | ^7-9^ |
| **Azithromycin** | *ermF* | ^10^ |
| **Azithromycin** | *ermG* | ^11^ |
| **Doxycycline** | *tetX* | ^12^ |
| **Doxycycline** | *tet32* | ^13^ |
| **Doxycycline** | *tet36* | ^14^ |
| **Doxycycline** | *tetB(P)* | ^15^ |
| **Doxycycline** | *tetQ* | ^16^ |
| **Doxycycline** | *tetT* | ^17^ |
| **Doxycycline** | *tetW* | ^18^ |
| **Doxycycline** | *tetM* | ^19^ |
| **Doxycycline** | *tetO* | ^20^ |
| **Doxycycline** | *tet44* | ^21^ |
| **Cefuroxime** | *cepA* | ^22^ |
| **Cefuroxime** | *cfxA*, *cfxA2*, *cfxA3*, *cfxA5*, *cfxA6* | ^23^ |
| **Ciprofloxacin** | *qepA* | ^24^ |

* AsRGs that not identified in the metagenome or virome were not listed in this table.

**Table S4. Phage contigs harboring antibiotic resistance genes (ARGs).**

| **Individual** | **Contig** | **ARG** | **AsRG**** | **# of Phage-like Contig** | **Host Species** |
| --- | --- | --- | --- | --- | --- |
| **AZY-a*** | contig-180_824 | *aph(3')-IIa* | Non-AsRG | 0 |  |
| **AZY-a** | contig-180_42 | *adeF* | Non-AsRG | 0 |  |
| **AZY-a** | contig-180_101 | *tet37* | Non-AsRG | 0 |  |
| **AZY-b** | contig-180_788 | *ermG* | AsRG | 3 |  |
| **AZY-b** | contig-180_1747 | *tetQ* | Non-AsRG | 2 |  |
| **AZY-b** | contig-180_1772 | *cfxA6* | Non-AsRG | 1 | *Prevotella copri* |
| **AZY-b** | contig-180_426 | *tet37* | Non-AsRG | 0 |  |
| **DOX-a** | contig-180_1841 | *cfxA6* | Non-AsRG | 1 | *Eubacterium hallii* |
| **DOX-a** | contig-180_285 | *tetQ* | AsRG | 10 | *Bacteroides caccae, Bacteroides fragilis* |
| **DOX-a** | contig-180_1736 | *tetW* | AsRG | 3 |  |
| **DOX-a** | contig-180_779 | *tet(W/N/W)* | Non-AsRG | 10 |  |
| **DOX-b** | contig-180_180 | *tet(W/N/W)* | Non-AsRG | 16 | *Bacteroides fragilis, Parabacteroides distasonis* |
| **DOX-b** | contig-180_1300 | *tetQ* | AsRG | 4 |  |
| **DOX-b** | contig-180_539 | *tet(W/N/W)* | Non-AsRG | 3 |  |
| **CFX-a** | contig-180_1047 | *tet(W/N/W)* | Non-AsRG | 3 |  |
| **CFX-a** | contig-180_4096 | *cfxA6* | AsRG | 1 |  |
| **CFX-a** | contig-180_2172 | *tet37* | Non-AsRG | 0 |  |
| **CFX-b** | contig-180_75 | *tetQ* | Non-AsRG | 11 | *Bacteroides fragilis, Blautia obeum, Bacteroides dorei* |
| **CFX-b** | contig-180_43 | *aadA13* | Non-AsRG | 9 | *Faecalibacterium prausnitzii* |
| **CIP-a** | contig-180_1537 | *tetQ* | Non-AsRG | 6 |  |
| **CIP-a** | contig-180_824 | *ermG* | Non-AsRG | 5 | *Bacteroides fragilis* |
| **CIP-b** | contig-180_681 | *adeF* | Non-AsRG | 1 |  |
| **CIP-b** | contig-180_953 | *tet(W/N/W)* | Non-AsRG | 22 |  |
| **CIP-b** | contig-180_4436 | *cblA-1* | Non-AsRG | 1 | *Bacteroides uniformis* |
| **CIP-b** | contig-180_2676 | *cepA* | Non-AsRG | 0 |  |
| **CIP-b** | contig-180_3714 | *cblA-1* | Non-AsRG | 3 |  |
| **CIP-b** | contig-180_6881 | *tetQ* | Non-AsRG | 2 | *Bacteroides fragilis* |
| **CIP-b** | contig-180_2744 | *ermG* | Non-AsRG | 1 | *Clostridium clostridioforme* |
| **CIP-b** | contig-180_24 | *tet37* | Non-AsRG | 0 |  |
| **CIP-b** | contig-180_9133 | *arnA* | Non-AsRG | 0 |  |
| **CTR-a** | contig-180_1072 | *aph(3')-IIa* | Non-AsRG | 0 |  |
| **CTR-b** | contig-180_425 | *cfxA5* | Non-AsRG | 4 | *Bacteroides ovatus, Dialister invisus, Bacteroides fragilis* |
| **CTR-b** | contig-180_528 | *tet(W/N/W)* | Non-AsRG | 44 | *Lachnospiraceae bacterium* |
| **CTR-b** | contig-180_350 | *tetQ* | Non-AsRG | 28 | *Clostridium bolteae, Capnocytophaga sp., Bacteroides fragilis* |
| **CTR-b** | contig-180_127 | *adeF* | Non-AsRG | 0 |  |
| **CTR-b** | contig-180_1294 | *ermG* | Non-AsRG | 6 |  |
| **CTR-b** | contig-180_6390 | *tetO* | Non-AsRG | 0 |  |
| **CTR-b** | contig-180_12 | *tet37* | Non-AsRG | 1 | *Bacteroides xylanisolvens* |

* AZY, azithromycin; DOX, doxycycline; CFX, cefuroxime; CIP, ciprofloxacin; CTR, control.

** AsRG, antibiotic-specific resistance gene homologous.

**Table S5. Antibiotic resistance gene (ARG) mobility assignments.**

| **Individual** | **Non-AsRG**: Non-Mobile** | **Non-AsRG: Mobile** | **Non-AsRG: Phage** | **Non-AsRG: Plasmid** | **Non-AsRG: TE***** | **AsRG: Non-Mobile** | **AsRG: Mobile** | **AsRG: Phage** | **AsRG: Plasmid** | **AsRG: TE** |
| --- | --- | --- | --- | --- | --- | --- | --- | --- | --- | --- |
| **AZY-a*** | 468 | 80 | 2 | 59 | 20 | 4 | 1 | 0 | 1 | 0 |
| **AZY-b** | 238 | 48 | 9 | 23 | 23 | 4 | 4 | 3 | 0 | 1 |
| **DOX-a** | 199 | 50 | 3 | 36 | 17 | 16 | 20 | 16 | 4 | 0 |
| **DOX-b** | 269 | 44 | 0 | 30 | 19 | 33 | 17 | 9 | 8 | 1 |
| **CFX-a** | 283 | 54 | 6 | 40 | 8 | 6 | 1 | 1 | 0 | 0 |
| **CFX-b** | 317 | 70 | 9 | 49 | 16 | 3 | 1 | 0 | 0 | 1 |
| **CIP-a** | 332 | 50 | 5 | 35 | 15 | 0 | 0 | 0 | 0 | 0 |
| **CIP-b** | 281 | 91 | 22 | 36 | 42 | 1 | 0 | 0 | 0 | 0 |

* AZY, azithromycin; DOX, doxycycline; CFX, cefuroxime; CIP, ciprofloxacin; CTR, control.

** AsRG, antibiotic-specific antibiotic resistance gene.

*** TE, transposable element. A mobile contig may be assigned to more than one category (phage-like contig, plasmid contig, and contig with TE).

**Titles of Supplemental Data (Title)**

**Data S1. Sequencing quality control.**

**a**. Metagenome, metatranscriptome, and virome libraries.

**b**. DNA library quality control.

**c**. RNA library quality control.

**d**. Phage library quality control.

**e**. Metagenomic co-assemblies.

**f**. Phage co-assemblies.

**Data S2. Species names used in this manuscript and different databases.**

**a**. Species names used by MetaPhlAn2, MiDAS, and the manuscript.

**b**. Species presented in the contig-species assignment (MiDAS) but not in the MetaPhlAn2 profile.

**c**. Species presented in the MetaPhlAn2 profile but not in the contig-species assignment (MiDAS).

**Supplemental References**

1. Truong DT, Franzosa EA, Tickle TL, Scholz M, Weingart G, Pasolli E, et al. MetaPhlAn2 for enhanced metagenomic taxonomic profiling. Nat Methods 2015; 12:902-3.

2. Emiola A, Oh J. High throughput in situ metagenomic measurement of bacterial replication at ultra-low sequencing coverage. Nat Commun 2018; 9:4956.

3. Ondov BD, Treangen TJ, Melsted P, Mallonee AB, Bergman NH, Koren S, et al. Mash: fast genome and metagenome distance estimation using MinHash. Genome Biol 2016; 17:132.

4. Altschul SF, Gish W, Miller W, Myers EW, Lipman DJ. Basic local alignment search tool. Journal of molecular biology 1990; 215:403-10.

5. Roux S, Faubladier M, Mahul A, Paulhe N, Bernard A, Debroas D, et al. Metavir: a web server dedicated to virome analysis. Bioinformatics 2011; 27:3074-5.

6. Roux S, Enault F, Hurwitz BL, Sullivan MB. VirSorter: mining viral signal from microbial genomic data. PeerJ 2015; 3:e985.

7. Yu L, Petros AM, Schnuchel A, Zhong P, Severin JM, Walter K, et al. Solution structure of an rRNA methyltransferase (ErmAM) that confers macrolide-lincosamide-streptogramin antibiotic resistance. Nat Struct Biol 1997; 4:483-9.

8. Min YH, Kwon AR, Yoon JM, Yoon EJ, Shim MJ, Choi EC. Molecular analysis of constitutive mutations in ermB and ermA selected in vitro from inducibly MLSB-resistant enterococci. Arch Pharm Res 2008; 31:377-80.

9. Hajduk PJ, Dinges J, Schkeryantz JM, Janowick D, Kaminski M, Tufano M, et al. Novel inhibitors of Erm methyltransferases from NMR and parallel synthesis. J Med Chem 1999; 42:3852-9.

10. Park AK, Kim H, Jin HJ. Phylogenetic analysis of rRNA methyltransferases, Erm and KsgA, as related to antibiotic resistance. FEMS Microbiol Lett 2010; 309:151-62.

11. Monod M, Mohan S, Dubnau D. Cloning and analysis of ermG, a new macrolide-lincosamide-streptogramin B resistance element from Bacillus sphaericus. J Bacteriol 1987; 169:340-50.

12. Yang W, Moore IF, Koteva KP, Bareich DC, Hughes DW, Wright GD. TetX is a flavin-dependent monooxygenase conferring resistance to tetracycline antibiotics. J Biol Chem 2004; 279:52346-52.

13. Melville CM, Scott KP, Mercer DK, Flint HJ. Novel tetracycline resistance gene, tet(32), in the Clostridium-related human colonic anaerobe K10 and its transmission in vitro to the rumen anaerobe Butyrivibrio fibrisolvens. Antimicrob Agents Chemother 2001; 45:3246-9.

14. Whittle G, Whitehead TR, Hamburger N, Shoemaker NB, Cotta MA, Salyers AA. Identification of a new ribosomal protection type of tetracycline resistance gene, tet(36), from swine manure pits. Appl Environ Microbiol 2003; 69:4151-8.

15. Roberts MC. Update on acquired tetracycline resistance genes. FEMS Microbiol Lett 2005; 245:195-203.

16. Leng Z, Riley DE, Berger RE, Krieger JN, Roberts MC. Distribution and mobility of the tetracycline resistance determinant tetQ. J Antimicrob Chemother 1997; 40:551-9.

17. Clermont D, Chesneau O, De Cespedes G, Horaud T. New tetracycline resistance determinants coding for ribosomal protection in streptococci and nucleotide sequence of tet(T) isolated from Streptococcus pyogenes A498. Antimicrob Agents Chemother 1997; 41:112-6.

18. Scott KP, Melville CM, Barbosa TM, Flint HJ. Occurrence of the new tetracycline resistance gene tet(W) in bacteria from the human gut. Antimicrob Agents Chemother 2000; 44:775-7.

19. Akhtar M, Hirt H, Zurek L. Horizontal transfer of the tetracycline resistance gene tetM mediated by pCF10 among Enterococcus faecalis in the house fly (Musca domestica L.) alimentary canal. Microb Ecol 2009; 58:509-18.

20. LeBlanc DJ, Lee LN, Titmas BM, Smith CJ, Tenover FC. Nucleotide sequence analysis of tetracycline resistance gene tetO from Streptococcus mutans DL5. J Bacteriol 1988; 170:3618-26.

21. Abril C, Brodard I, Perreten V. Two novel antibiotic resistance genes, tet(44) and ant(6)-Ib, are located within a transferable pathogenicity island in Campylobacter fetus subsp. fetus. Antimicrob Agents Chemother 2010; 54:3052-5.

22. Rogers MB, Parker AC, Smith CJ. Cloning and characterization of the endogenous cephalosporinase gene, cepA, from Bacteroides fragilis reveals a new subgroup of Ambler class A beta-lactamases. Antimicrob Agents Chemother 1993; 37:2391-400.

23. Parker AC, Smith CJ. Genetic and biochemical analysis of a novel Ambler class A beta-lactamase responsible for cefoxitin resistance in Bacteroides species. Antimicrob Agents Chemother 1993; 37:1028-36.

24. Yamane K, Wachino J, Suzuki S, Kimura K, Shibata N, Kato H, et al. New plasmid-mediated fluoroquinolone efflux pump, QepA, found in an Escherichia coli clinical isolate. Antimicrob Agents Chemother 2007; 51:3354-60.
